# Aging Influence on Gray Matter Structural Associations within the Default Mode Network Utilizing Bayesian Network Modeling

**DOI:** 10.3389/fnagi.2014.00105

**Published:** 2014-05-30

**Authors:** Yan Wang, Kewei Chen, Jiacai Zhang, Li Yao, Ke Li, Zhen Jin, Qing Ye, Xiaojuan Guo

**Affiliations:** ^1^College of Information Science and Technology, Beijing Normal University, Beijing, China; ^2^Banner Alzheimer’s Institute and Banner Good Samaritan PET Center, Phoenix, AZ, USA; ^3^State Key Laboratory of Cognitive Neuroscience and Learning, Beijing Normal University, Beijing, China; ^4^Laboratory of Magnetic Resonance Imaging, The 306th Hospital of People’s Liberation Army, Beijing, China

**Keywords:** normal aging, Bayesian network modeling, default mode network, structural associations, gray matter

## Abstract

Recent neuroimaging studies have revealed normal aging-related alterations in functional and structural brain networks such as the default mode network (DMN). However, less is understood about specific brain structural dependencies or interactions between brain regions within the DMN in the normal aging process. In this study, using Bayesian network (BN) modeling, we analyzed gray matter volume data from 109 young and 82 old subjects to characterize the influence of aging on associations between core brain regions within the DMN. Furthermore, we investigated the discriminability of the aging-associated BN models for the young and old groups. Compared to their young counterparts, the old subjects showed significant reductions in connections from right inferior temporal cortex (ITC) to medial prefrontal cortex (mPFC), right hippocampus (HP) to right ITC, and mPFC to posterior cingulate cortex and increases in connections from left HP to mPFC and right inferior parietal cortex to right ITC. Moreover, the classification results showed that the aging-related BN models could predict group membership with 88.48% accuracy, 88.07% sensitivity, and 89.02% specificity. Our findings suggest that structural associations within the DMN may be affected by normal aging and provide crucial information about aging effects on brain structural networks.

## Introduction

Normal aging is typically accompanied by progressive and gradual decline in memory and executive control functions together with morphological changes in the brain (Damoiseaux et al., 2008; Miller et al., 2008; Madden et al., 2010). A number of structural magnetic resonance imaging (MRI) studies have shown that normal aging-related morphological changes involve significant reductions in gray matter volume or cortical thickness (Good et al., 2001; Taki et al., 2004; Smith et al., 2007; Kalpouzos et al., 2009). Moreover, most of these studies have consistently depicted a common pattern of gray matter atrophy in the prefrontal cortex (Raz et al., 1997; Tisserand et al., 2002; Lemaitre et al., 2012) and the medial temporal lobe (Sullivan et al., 1995; Lemaitre et al., 2012). Interestingly, many important brain regions with high centrality (hubs) were located in the prefrontal regions, and these aging-affected regions were associated with deficits in cognitive functions (Bullmore and Sporns, 2009; Wu et al., 2012).

Most of the previous structural MRI studies focused on localizing brain regions using univariate statistical approaches, and they might have potentially missed the covariant morphometric information related to normal aging. Using multivariate analytical methods, several investigations have revealed the brain structure’s small-world attributes [characterized by high degrees of local clustering among regions-of-interest (ROIs) and short paths linking all ROIs] or modularity (defined by distinct ROI groups with dense connections within each ROI-group and sparse connections between these ROI groups) (Bullmore and Sporns, 2009). These publications suggested that normal aging-related changes exhibited the organized inter-regional covariance of morphological features as a well-defined network (Bergfield et al., 2010; Chen et al., 2011; Montembeault et al., 2012). In addition, the scaled subprofile model (SSM) was also used to depict an age-related structural network, which showed concurrent decreases in gray matter volume, notably in the bilateral medial frontal, insula/perisylvian, and anterior cingulate regions (Bergfield et al., 2010). Finally, utilizing cortical thickness correlation analysis, Chen et al. (2011) found that aging was associated with organizational alterations of structural networks. Although these recent MRI studies have constructed structural networks associated with normal aging, less is known about the influence of aging on inter-region dependencies among spatially distributed regions within such brain networks.

On top of the above-mentioned structural networks, the human brain is also intrinsically organized into complicated functional networks (Bullmore and Sporns, 2009; Bassett and Gazzaniga, 2011; Sporns, 2011). It has been widely accepted that brain activity in resting state is organized into several functionally relevant networks (De Luca et al., 2006; Mantini et al., 2007) such as the default mode network (DMN), attention and visual/auditory networks. Literature findings suggest that these resting state functional networks are related to structural networks, therefore providing crucial insight into structural networks (or vice versa).

Among these functional networks, the DMN, with its core regions such as the posterior cingulate cortex (PCC) and medial prefrontal cortex (mPFC), is one of the most frequently discussed networks. For example, using a combination of diffusion tensor imaging (DTI) and resting state functional MRI, researchers have demonstrated that functional connectivity in resting state reflects structural connectivity within the DMN (Damoiseaux and Greicius, 2009; Greicius et al., 2009). Furthermore, normal aging was also related to alterations in functional connectivity in the DMN, especially decreased connectivity among the PCC, mPFC, and parietal cortex (Hafkemeijer et al., 2012). Additionally, using cortical thickness or gray matter volume, some researchers have indicated that old adults have distinctly reduced intra-module connections in the DMN when compared with young adults (Chen et al., 2011; Wu et al., 2012). Together, these findings suggested the needs for further exploring aging-associated alterations in the DMN in the context of the structural network organizational changes.

The organizational changes could be investigated using Bayesian network (BN) approach, which was introduced and utilized in neuroimaging studies (Zheng and Rajapakse, 2006). Without a prior model configuration, BN modeling can be used to investigate association dependency, or directed connection, of one ROI on another. Here, the directed connection is in the context of conditional probability (Chen and Herskovits, 2006; Zheng and Rajapakse, 2006). As a tool to investigate associations among variables, such as ROIs in neuroimaging studies, BN approach has been successfully applied to study functional networks in Alzheimer’s disease (AD) (Wu et al., 2011) and structural networks in mild cognitive impairment (MCI) based on MRI (Chen and Herskovits, 2006; Chen et al., 2012). These findings indicated that BN approach was capable of characterizing associations among brain regions. The feasibility of using BN approach to investigate the effect of normal aging on structural networks, such as the DMN, is however not well documented in the literature.

Using BN approach and structural MRI data from healthy young and old subjects, the current study aimed to explore the influence of aging on associations of regional gray matter volume among the core DMN regions. These structural associations are in terms of probabilistic dependence. The association differences between these two groups were assessed statistically by using a non-parametric permutation test. Finally, we investigated the discriminability of aging-associated BN models to classify young and old subjects.

## Materials and Methods

### Subjects

All participants in this study were from the Open Access Series of Imaging Studies (OASIS) database^1^ including 109 young adults [22.73 ± 2.34 years old (range: 20–28), 65 females, and 44 males] and 82 healthy old adults [74.37 ± 8.23 years old (range: 60–90), 60 females, and 22 males]. Young subjects were recruited from the Washington University community and questioned about their medical histories and use of psychoactive drugs. Older adults were recruited from the Washington University’s Alzheimer Disease Research Center (ADRC) and underwent ADRC’s full assessment (Marcus et al., 2007). The dementia status of old adults was assessed by Mini-Mental State Examination (MMSE) (Folstein et al., 1975) and Clinical Dementia Rating (CDR) scores (Morris, 1993). In this study, the healthy old subjects (CDR = 0) had mean MMSE scores of 29.02 ± 1.27 (range: 25–30). The young group did not differ from the old group in sex ratio $\left(\chi_{1}^{2}=3.792,\;p=0.051\right)$. All subjects participated in accordance with guidelines of the Washington University Human Studies Committee. The detailed demographics of all participants were described in Marcus et al. (2007) report.

### MRI acquisition

For each subject, three or four sagittally T1-weighted MPRAGE images were collected on a 1.5-T MRI scanner (TR/TE/TI = 9.7/4.0/20 ms, flip angle = 10°, FOV = 256 mm × 256 mm, voxel size = 1 mm × 1 mm, slices = 128, thickness = 1.25 mm). For the sake of increasing signal-to-noise ratio, the T1 image selected in this study for each subject was a motion-corrected coregistered average image (1 mm × 1 mm × 1 mm) of all available data (Marcus et al., 2007).

### Image pre-processing

All of the structural T1 images were pre-processed using the VBM8 Toolbox^2^ in SPM8^3^. Using adaptive maximum posterior and partial volume estimation (Rajapakse et al., 1997; Tohka et al., 2004), the structural image for every subject was segmented into rigid-body aligned gray matter, white matter, and cerebrospinal fluid (CSF) maps. Two denoising methods, the spatially adaptive non-local means denoising filter and classical Markov Random Field approach, were applied to improve the image segmentation. The gray matter image was normalized to the Montreal Neurological Institute (MNI) space by high dimensional diffeomorphic anatomical registration using exponential Lie algebra (DARTEL) algorithm (Ashburner, 2007). DARTEL parameterizes diffeomorphic and inverse consistent deformations using a time-invariant velocity field. The normalized gray matter maps were modulated by Jacobian determinants from the deformations to preserve the total amount of tissue in the native spaces. Finally, all of the gray matter maps were smoothed with a Gaussian kernel of 8 mm full width at half maximum (FWHM).

### ROIs definition

We selected eight core ROIs based on previous studies (Fox et al., 2005; Fair et al., 2008). Table 1 shows the names and corresponding abbreviations of these eight ROIs. Each ROI mask was generated by using the WFU_PickAtlas software^4^ (Maldjian et al., 2003). Every ROI covered the entire area of the corresponding anatomical region defined by the AAL atlas. We defined gray matter volume of each ROI for each individual as an average value of all voxel intensity above 0.15 cut-off value within the ROI. The average gray matter volumes of the eight ROIs were entered into the BN model as continuous variables to construct structural associations within the DMN for the young and old adult groups.

**Table 1:** **Brain regions and the corresponding abbreviations of eight ROIs in the DMN**.

Brain regions	Abbreviations
Posterior cingulate cortex	PCC
Medial prefrontal cortex	mPFC
Left hippocampus	lHP
Right hippocampus	rHP
Left inferior parietal cortex	lIPC
Right inferior parietal cortex	rIPC
Left inferior temporal cortex	lITC
Right inferior temporal cortex	rITC

### Bayesian network analysis

Bayesian network model is a directed acyclic graph (DAG) used to describe conditional dependence among nodes. For two nodes *x*_1_ and *x*_2_ in a DAG, the directed arc from *x*_1_ to *x*_2_ represents the probabilistic dependence of *x*_2_ on *x*_1_ (Chen and Herskovits, 2006; Zheng and Rajapakse, 2006). In the context of the conditional probability concept, this is often depicted as the influence of parent node *x*_1_ on child node *x*_1_. In our study and many others using BN modeling (Chen and Herskovits, 2006; Wu et al., 2011; Chen et al., 2012), the probabilistic dependence of one brain region on another is phrased as a “direction” from one brain region to another.

In this study, for each group, eight ROIs were regarded as nodes of BN model and a set of average gray matter volumes of ROIs were used as continuous variables that were entered into the model. To generate an optimal BN model, we applied the popular search-and-score approach to acquire graph structure and used a maximum likelihood estimation (MLE) procedure to obtain parameters (Zheng and Rajapakse, 2006). The search-and-score approach (Chickering, 2003), using the Bayesian Information Criterion (BIC) score, searches and assesses all the possible DAGs by adding and removing edges between any two nodes until returns the one with the highest score. The BIC is described as follows:

$$\text{BIC}\left(\theta \right)=\sum_{j=1}^{d}L\left(j,\pi_{j},{\hat{\theta }}_{j}^{\text{mle}}\right)-\frac{\left|{\hat{\theta }}_{j}^{\text{mle}}\right|}{2}\log n$$

where *d* is the number of nodes or ROIs, and *n* indicates the number of the sample; the *j*^th^ expression $L\left(j,\pi_{j},\theta \right)=\sum_{i=1}^{n}\log p\left(X_{i,j}∕X_{i,\pi_{j}},\theta \right)$ in the summation is the log-likelihood of node *j*, indicating the fitness degree of the model to the data; the term $\frac{\left|{\hat{\theta }}_{j}^{\text{mle}}\right|}{2}\log n$ in the BIC formula above is the penalty on the model complexity; ${\hat{\theta }}_{j}^{\text{mle}}=\arg\;\sup_{\theta }L\left(j,\pi_{j},\theta \right)$ is the MLE of the parameter of node *j*. All of the procedures were implemented with the Bayesian Net Toolbox^5^ in MATLAB R2010.

After constructing the BN model, the conditional probability density can be calculated for node *j* given its parent node-set π*_j_*, which directly connects with node *j* in the graph structure:

$$\begin{array}{llll} & p\left(X_{j}\left|\pi_{j},\theta_{j}\right.\right)\; & & \;\\ & \;=\frac{1}{{\left(2\pi \right)}^{1∕2\;}{\left|\Sigma \right|}^{1∕2\;}}\exp\left\{-\frac{1}{2}{\left(X_{j}-\mu_{j}\right)}^{T}\Sigma^{-1}\left(X_{j}-\mu_{j}\right)\right\}\; & & \; \end{array}$$

where μ*_j_* and Σ*_j_* respectively represent the conditional mean and variance of *X_j_*; θ*_j_* is the parameter for node *j*. Then, the joint probability density for all of the ROIs can be described as $p\left(X\right)=\prod_{j=1}^{d}p\left(X_{j}|\pi_{j},\theta_{j}\right)$.

A non-parametric permutation test with 5000 permutations on the distributions of all subjects was employed to detect the differences in all connection weight coefficients between two BN models. Here, the connection is about the existence of an edge between two brain regions in the BN models and represents a statistical association between the corresponding variables. And the weight coefficient represents the strength of structural association or the volumetric correlation strength. In the end, we assessed the significance of the difference by calculating the type-I error probability of Young > Old or Old > Young.

Additionally, we used the joint probability density values to assign the membership of a subject to one of the two groups and used the receiver operating curve (ROC) to assess the discriminability.

## Results

### Structural associations within the DMN

**Figure 1:**
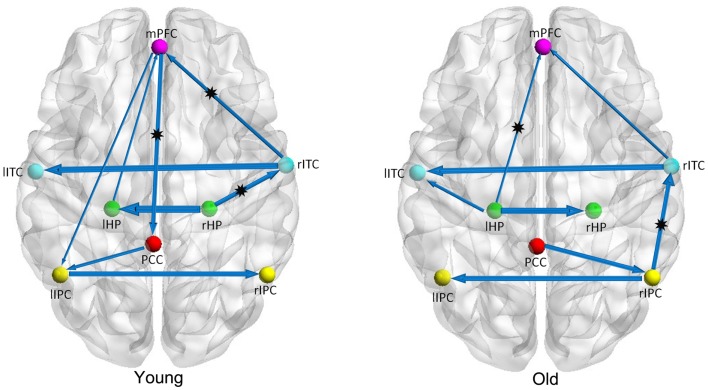
**Bayesian network models of the DMN in the young (left panel) and old (right panel) groups**. The arrows represent dependencies among brain regions and the thickness of the arrows is proportional to the strength of the connections. The asterisks indicate the connections that were significantly stronger in young/old than in old/young groups.

Figure 1 shows two BN models for the young and old groups, representing structural associations or probabilistic dependence among the core DMN regions. Table 2 lists the corresponding connection directions and weight coefficients. Connections lHP → mPFC, rITC → lITC, and rITC → mPFC were present in both young and old groups, connections rHP → rITC, PCC → lIPC, mPFC → PCC, and mPFC → lIPC were observed only in the young group, and connections lITC → lHP, rIPC → rITC, and PCC → rIPC were observed only in the old group. Although connections between rHP and lHP, rIPC and lIPC were revealed in both groups, they were opposite in direction.

**Table 2:** **List of connections and the corresponding weight coefficients in the Bayesian network models of the young and old groups**.

Connections		Weight coefficients of connections	
		Young	Old
I	lHP → mPFC	0.284	0.292
	rITC → lITC	0.784	0.723
	rITC → mPFC	0.469	0.372
II	rHP → rITC	0.576	
	PCC → lIPC	0.422	
	mPFC → PCC	0.548	
	mPFC → lIPC	0.292	
III	lITC → lHP		0.425
	rIPC → rITC		0.559
	PCC → rIPC		0.576
IV	rHP → lHP	0.921	
	lHP → rHP		0.773
	rIPC → lIPC		0.649
	lIPC → rIPC	0.625	

*Part I of this table lists connections that exist in both groups. Parts II and III show the connections present only in the young and old groups, respectively. Part IV lists connections that are opposite in direction in two groups*.

### Between-group association differences

We adopted a permutation test to investigate between-group differences in the connection weight coefficients with the probabilities of type-I errors listed in Table 3. The column “Young > Old” shows the probabilities of type-I errors under the null hypothesis that the strength of structural association in the young group is stronger than in the old group, but the column “Old > Young” displays the opposite. The connections rITC → mPFC, rHP → rITC, and mPFC → PCC were stronger in the young group than in the old group, which were determined by a significance level of uncorrected *p* < 0.05. Using the same assessment, the lHP → mPFC and rIPC → rITC connections showed significantly decreased alterations in the young group when compared with the old group.

**Table 3:** **The probabilities of Type-I errors for between-group differences in all connections**.

Young > Old		Old > Young	
Connections	Probabilities	Connections	Probabilities
lHP → mPFC	1.000	**lHP → mPFC**	**0.000**
rITC → lITC	0.334	rITC → lITC	0.666
**rITC → mPFC**	**0.000**	rITC → mPFC	1.000
**rHP → rITC**	**0.041**	lITC → lHP	0.064
PCC → lIPC	0.150	**rIPC → rITC**	**0.000**
**mPFC → PCC**	**0.050**	PCC → rIPC	0.832
mPFC → lIPC	0.220	lHP → rHP	0.145
rHP → lHP	0.232	rIPC → lIPC	0.236
lIPC → rIPC	0.233		

*The column “Young > Old” displays the probabilities of type-I errors in the hypothesis that the strength of connections in the young group is stronger than in the old group. The other column “Old > Young” shows the opposite. The probabilities marked in bold indicate significantly stronger connections (*p* < 0.05)*.

### Classification ability

The classification results for the young versus the old groups are summarized as follows. The classification accuracy based on the derived two BN models reached 88.48%, and the corresponding specificity and sensitivity were 89.02 and 88.07%, respectively.

## Discussion

In the present study, we applied BN method to characterize gray matter associations among core brain regions within the DMN of young and old adults. Then, we employed a non-parametric permutation test to detect the BN connection differences in weight coefficients between two groups. Furthermore, we evaluated the discriminability of the aging-related BN models by comparing joint probability density scores based on the BN models in each of the young and old groups. The permutation test showed significant reductions in the connections rITC → mPFC, rHP → rITC, and mPFC → PCC and increases in the connections lHP → mPFC and rIPC → rITC in the old group when compared with the young group. In addition, the aging-related BN models could predict the membership of subjects with high accuracy, sensitivity, and specificity.

In contrast to the BN model in young adults, the one in old adults revealed some coordination disruptions among the DMN core regions, possibly due to aging. Our findings, in this regard, are consistent with several published studies. For example, using correlation analysis, previous studies based on cortical thickness or gray matter volume consistently demonstrated that intra-modular connections in the DMN in the old group were more reduced than in the young group (Chen et al., 2011; Wu et al., 2012). Additionally, Hafkemeijer et al. (2012) summarized various age-related studies of brain function and found that the DMN generally showed reduced functional connectivity as a consequence of the normal aging process. Decreased functional connectivity might reflect structural alteration of brain network.

Our findings revealed that there were strong structural associations between brain regions in one hemisphere and the homologous regions in the opposite hemisphere in both young and old groups. However, the strengths of structural associations were generally greater in the young group than in the old group. The decreased strengths of structural associations between two homologous brain regions reflected inconsistency in the degree of atrophy between right and left hemispheres in the old group. Some previous studies attempted to explore the possible causes for the reduced homologous inter-hemisphere connections. For example, Vernooij et al. (2008) found white matter atrophy in corpus callosum might lead to a decrease in structural connections between bilateral brain regions. In another study, Mechelli et al. (2005) proposed that the gray matter density of a brain region could predict the density of a homologous region located in the opposite hemisphere. In addition to the fact that corpus callosum contributed to the inter-hemispheric structural connectivity, a previous study demonstrated that loss of corpus callosum integrity affected functional connectivity (Quigley et al., 2003).

The prominent between-group changes in the strengths of structural associations included the decreased connections from rITC to mPFC, rHP to rITC, and mPFC to PCC and increased connections from lHP to mPFC and rIPC to rITC in the old group compared with the young group. The prefrontal cortex is well known to be associated with executive control function (Madden et al., 2010). Overall, in our study, the number of connections with mPFC in the old group was reduced when compared with the young group. In a previous DTI study of normal aging, Grieve et al. (2007) found that prefrontal regions showed notable negative relationship with age, which could be a factor that affected the strengths of structural associations between other regions and mPFC as proposed in our study. Another study, based on seed-ROI, verified that the functionally correlated DMN regions were also volumetrically correlated and that such correlations between the right angular cortex and some frontal regions were significantly decreased in the old group (Montembeault et al., 2012). This observed alteration is consistent with our present result showing connections between mPFC and lIPC present only in the young group. Moreover, Vernooij et al. (2008) found that fractional anisotropy in cingulate bundle was reduced, which might result in decreased structural associations between mPFC and PCC found in the current study. Andrews-Hanna et al. (2007) also reported that older adults showed decreased functional connectivity between mPFC and PCC. Additionally, using cortical thickness, Chen et al. (2011) showed that the correlation between right mPFC and left precuneus was decreased due to aging, which is also consistent with our findings. Pertaining to increased structural associations in the old group, it was possible that these results were due to a connectivity compensation, a concept described as some brain regions working harder to make up for the deficiencies of other regions in the network (Cappell, 2008).

Additionally, we noted that a small number of connections (between lHP and rHP, between lIPC and rIPC) had reversal direction in the two groups but showed no statistically significant between-group differences. We speculated that the direction alternation might be influenced by aging among other factors. The explanation on the direction reversal should be with great caution since the association dependency is in terms of the conditional probability (Chen and Herskovits, 2006). More importantly, Smith et al. (2011) suggested that it was more difficult to achieve accurate estimation of connection directionality by BN approach in spite of its high sensitivity of detecting the presence of connections.

Bayesian network modeling can be used to examine probabilistic associations among variables. Till now, BN approach has been successfully utilized in neuroimaging (including functional MRI and structural MRI) studies (Chen and Herskovits, 2006; Zheng and Rajapakse, 2006; Wu et al., 2011; Chen et al., 2012). For functional MRI data, BN modeling examines conditional dependencies of brain activity based on functional MRI time series for each individual subject. For structural MRI data, BN modeling investigates probabilistic associations of morphological feature based on morphometric variables such as gray matter volume from all subjects at the group level either within or between groups. A number of publications have suggested that brain regions covary in their morphological properties, and such structural networks coordinate due to various factors such as normal aging (Bergfield et al., 2010; Chen et al., 2011; Montembeault et al., 2012). Furthermore, some previous studies proposed that structural covariances may result from mutually trophic influences or common experience-related plasticity that are mediated by white matter connections (Ferrer et al., 1995; Mechelli et al., 2005; Soriano-Mas et al., 2013) and the altered relation between regions may arise from lack of mutually trophic influences in different clinical conditions (He et al., 2008; Seeley et al., 2009). Therefore, BN modeling, as a valuable method of mining association relationships between continuous variables, can be used to investigate the association dependency based on regional gray matter volumes. Nevertheless, our current results on the directional relationship are statistical in nature and they cannot replace direct biological and medical evidence. We hope that our study provides additional, consistent but preliminary findings in support of more comprehensive investigations in this regard.

In addition to examining the network differences, we employed the BN model as a classification tool to infer group membership of subjects by comparing the joint probability densities between the young and the old groups. This operation for classification integrated all gray matter volume information from eight ROIs instead of focusing on obvious morphological changes in some particular brain regions. The ROC analysis demonstrated the discriminability of the BN model with 88.07% sensitivity, 89.02% specificity, and 88.48% accuracy. Both the sensitivity and specificity were close to 90%, which not only verified the validity of our age-associated BN models but also provided an evidence for the BN model to server as a predictive brain biomarker in structure for normal aging.

Although the accuracy of classification was close to 90%, we noted that we examined the discriminability of the derived BN models by comparing joint probability densities of the subjects used to construct BN models. This classification of *post hoc* nature has limited validity. Thus, its generalizability needs to be cross-validated using independent dataset. Additional studies are needed to verify the replicability and stability of the aging-related BN models in an independent dataset.

In summary, our study suggests that structural associations within the DMN are affected by the normal aging process. The BN modeling approach potentially can serve as a useful tool for studying structural associations or probabilistic dependence among multiple brain regions.

## Conflict of Interest Statement

The authors declare that the research was conducted in the absence of any commercial or financial relationships that could be construed as a potential conflict of interest.

## Footnotes

^1^http://www.oasis-brains.org

^2^http://dbm.neuro.uni-jena.de/vbm8

^3^http://www.fil.ion.ucl.ac.uk/spm

^4^http://www.ansir.wfubmc.edu

^5^https://code.google.com/p/bnt/
